# Users’ perception of quality as a driver of private healthcare use in Mexico: Insights from the People’s Voice Survey

**DOI:** 10.1371/journal.pone.0306179

**Published:** 2024-06-25

**Authors:** Svetlana V. Doubova, Hannah H. Leslie, Ricardo Pérez-Cuevas, Margaret E. Kruk, Catherine Arsenault

**Affiliations:** 1 Epidemiology and Health Services Research Unit CMN Siglo XXI, Mexican Institute of Social Security, Mexico City, Mexico; 2 Division of Prevention Science, University of California San Francisco, San Francisco, California, United States of America; 3 Division of Social Protection and Health, Interamerican Development Bank, Washington, DC, United States of America; 4 Department of Global Health and Population, Harvard T.H. Chan School of Public Health, Boston, MA, United States of America; 5 Department of Global Health, Milken Institute School of Public Health, The George Washington University, Washington, DC, United States of America; Public Library of Science, UNITED KINGDOM

## Abstract

**Objective:**

The Mexican government has pursued multiple initiatives to improve healthcare coverage and financial protection. Yet, out-of-pocket health spending and use of private sector providers in Mexico remains high. In this paper, we sought to describe the characteristics of public and private healthcare users, describe recent visit quality across provider types, and to assess whether perceiving the public healthcare sector as poor quality is associated with private health sector use.

**Methods and findings:**

We analyzed the cross-sectional People’s Voice Survey conducted from December 2022 to January 2023. We used Chi-square tests to compare contextual, individual, and need-for-care factors and ratings of most recent visits between users of public (social security and other public providers) and private sector providers (stand-alone private providers and providers adjacent to pharmacies). We used a multivariable Poisson regression model to assess associations between low ratings of public healthcare sources and the use of private care. Among the 811 respondents with a healthcare visit in the past year, 31.2% used private sources. Private healthcare users were more educated and had higher incomes than public healthcare users. Quality of most recent visit was rated more highly in private providers (70.2% rating the visit as excellent or very good for stand-alone private providers and 54.3% for pharmacy-adjacent doctors) compared to social security (41.6%) and other public providers (46.6%). Those who perceived public health institutions as low quality had a higher probability of seeking private healthcare.

**Conclusion:**

Users rated public care visits poorly relative to private care; at the population level, perceptions of poor quality care may drive private care use and hence out-of-pocket costs. Improving public healthcare quality is necessary to ensure universal health coverage.

## Introduction

In low- and middle-income countries (LMIC), the private healthcare sector is a critical stakeholder in health service delivery [1]. Country-specific evidence on the characteristics and performance of the private healthcare sector can inform national policy towards ensuring that healthcare services are accessible, affordable, equitable, and high quality [2]. Prior research across LMIC has found that private providers, while highly heterogeneous [1–3], are sometimes more user-centered and have more consistent equipment availability than public healthcare providers, while public providers can be more efficient, providing less potentially unnecessary testing and treatment, and linked to better patient outcomes [3, 4]. Studies specific to Latin American countries have found higher satisfaction, shorter wait times, and better medication access within private health services, although availability of recent data is limited [5–8].

Mexico’s healthcare system is complex, fragmented, and inequitable. Approximately 65% of the country’s 130 million people are affiliated with social security institutions that offer comprehensive economic, health, and social benefits to formal sector workers and their families [9]. Social security insitutions include the Instituto Mexicano de Seguridad Social (IMSS), the Instituto de Seguridad y Servicios Sociales de Trabajadores del Estado and social security institutions for oil, army and navy workers. Formal sector workers support financing of these plans through a payroll tax. Most of the remaining population is uninsured [10]. Uninsured individuals tend to have a lower socioeconomic status and less education than social security affiliates, and they rely on either public programs such as the IMSS-Bienestar program, or on the private health care sector. IMSS-Bienestar provides no-cost basic healthcare, but it does not provide care for chronic conditions requiring tertiary care services like cancer [11].

The private healthcare sector consists of independent and corporate providers and not-for-profit organizations. Two thirds of hospitals and over half of outpatient clinics are private [12]. In addition, there are approximately 15,000 doctors adjacent to, and employed by, pharmacies that provide ambulatory care [13]. Private healthcare is estimated to account for over half of care seeking in Mexico, and it only increased during the COVID-19 pandemic [10, 14, 15]. Most private sector services are paid out-of-pocket, as private insurance is uncommon (<10% of the population) and primarily covers hospital services [16]. In 2019, 42.1% of total health expenditure was out-of-pocket [17].

Research from the 2000s suggests that perceptions of better quality, shorter wait times, and better health outcomes in the private sector compared to the public sector contributed to higher rates of private healthcare utilization despite the cost [18, 19]. However, private pharmacy-adjacent doctors may provide substandard care by overprescribing medications and failing to comply with government regulations [20, 21]. Care quality is one of several individual and contextual factors shaping the choice of provider [22–24]. Several studies from the United States and Europe identified that users rely on their previous healthcare experiences when deciding where to receive healthcare [24], and confidence in government and healthcare providers more broadly is associated with greater intended health service uptake [25]. A recent (2022) study in Mexico found that 56% of the population that required healthcare in the last three months sought care in private health facilities, despite having the right to receive public healthcare. Out of those who chose private healthcare providers, 21% reporting doing so because of poor quality in public health facilities, such as long waiting times and unkind staff, while the rest cited access or other barriers [26]. Nevertheless, evidence on the role of the quality of public and private systems in shaping users’ choice of healthcare provider remains scarce [24, 27].

In this study, we aimed to (1) characterize private and public healthcare users based on individual and contextual factors, (2) compare users’ perceived quality of care between public and private providers, and (3) assess whether the perception of low quality of public healthcare services is associated with a greater probability of use of private healthcare.

## Materials and methods

We performed secondary data analysis of the cross-sectional People’s Voice Survey conducted in Mexico from December 2022 to January 2023. This survey was developed by the QuEST Network, a global consortium for research on high-quality health systems (https://questnetwork.org) [28], and undertaken by the market research firm SSRS. The survey content was informed by the 2018 framework on high-quality health system domains and components [27, 28]. The survey applied random probability sampling to obtain a nationally representative sample of adults 18 years and older using a random digit dialing (RDD) approach from overlapping mobile and fixed phone frames. An RDD framework was built using the national numbering plan provided by Federal Telecommunications Institute. Based on the numbering plan, Sampling Solutions International developed a probabilistic design for pulling “seed” blocks from which actual phone numbers were generated, stratified by region according to the population distribution. The survey response rate was 3%. The survey sample included 1002 participants. Post-stratification weights were constructed based on demographic variables to account for differences in sample design and probability of selection. The final sample is, therefore, statistically projectable to the adult population in Mexico.

An expert group of the QuEST network designed, validated, and translated to Spanish the study questionnaire and adapted it for the Mexican context. Before the fieldwork, a pilot test of the questionnaire was performed to ensure language clarity. SSRS programmed the questionnaire using Computer Assisted Telephone Interviewing software.

### Study population

To address the study objectives, our analytic sample is limited to respondents who reported using healthcare services during the last year and specified the healthcare provider used for their last visit.

### Ethics

The present study analyzed anonymized data from the People’s Voice Survey that were accessed for research purposes on March 15, 2023. The market research firm SSRS undertook patients’ invitation to the study and obtained verbal consent to participate previous to the survey application. This procedure was approved by the Harvard University Institutional Review Board that deemed the People’s Voice Survey research exempt from full review.

### Study variables

We classified users into four groups based on the type of provider used for their last healthcare visit: stand-alone private providers, private pharmacy-adjacent doctors, social security healthcare providers, and public healthcare providers for those without social security.

We drew from the Anderson model of health services utilization to characterize individuals within each group [22, 23]. Based on items available in the survey, we considered the following individual factors: (a) predisposing (gender, age [18–44 years, 45–64 years, 65+ years], educational attainment [≤9 years and ≥10 years]; (b) enabling characteristics (monthly household income [low income ≤ 10,000 pesos/month, middle and high income >10,000 pesos/month], health insurance affiliation [no insurance, private health insurance and social security], having a usual source of care); (c) need for care, including presence of chronic disease [yes/no], self-rated health [excellent/very good/good, fair/poor] and having an unmet health need in the last year. The contextual factors included area of residence [rural, urban] and three region groups based on the average household income in the state [poorest, middle, richest].

To summarize perceived healthcare quality, we assessed ratings of the most recent visit using ten indicators, each measured on a five-point Likert scale ranging from “poor” to “excellent”: (1) assessment of overall quality of care, (2) provider’s knowledge and skills, (3) availability of equipment and other supplies, (4) respectful attitude, (5) knowledge about patient’s previous consultations and the results of his/her tests, (6) clarity of information provided, (7) patient involvement in making decisions about healthcare, (8) wait time, (9) consultation time, and (10) kindness and supportive attitude of the rest of the health facility staff. Each indicator was coarsened to 3 categories [excellent/very good, good, and fair/poor].

Our analysis also included the overall perception of the quality of healthcare in public healthcare institutions. The People’s Voice Survey assessed perceptions of health system quality by asking all respondents, ‘Overall, how would you rate the quality of the [specify] system in your country?’ with the item repeated for Social Security, Ministry of Health (MoH), and IMSS Bienestar systems. The responses for these questions were on a five-point Likert scale (ranging from “poor” to “excellent”). We used the responses to these three questions to create a binary variable to determine whether the respondent perceived fair or poor quality in any of these three public healthcare institutions.

### Statistical analysis

We conducted descriptive analyses to characterize private and public healthcare users and summarize perceived quality by source of last visit, reporting proportions for each variable and using chi-square tests to detect statistically significant (p<0.05) deviations from expected frequencies. We used survey weights to account for the survey design.

To assess whether the perception of low quality of public healthcare services is associated with a higher likelihood of private sector use, we excluded from the analysis users with private health insurance (n = 49), focusing on those without private insurance, who would incur out-of-pocket expenses for services that might have been available through public sources (n = 762). We performed multivariable robust Poisson regression to control for individual and demographic factors [29]. We performed double weighting (with survey and missing data stabilized inverse probability weights) to avoid bias related to the missing data in the responses of the survey participants, as 13.2% of them had missing data in one or more study variables. The analysis comprised the following steps: First, we generated stabilized inverse probability weights [30, 31]. The denominator for stabilized inverse probability weights was the probability of having missing data given the available covariates without missing data. The covariates were the participants’ sex, age, educational attainment, and chronic disease. The numerator was the probability of having missing data regardless of the covariates. Then, we created combined weights by multiplying stabilized inverse probability weights and survey weights to ensure that the analysis was generalizable to the survey target population. Finally, we fitted a multivariable Poisson regression to estimate rate ratios as an approximate to relative risk (probability) of using private healthcare by including in the analysis all conceptually relevant confounders related to the study outcome [32], as defined by the Anderson conceptual model and previous studies in this field. Prior to performing the multiple regression analyses, we confirmed the absence of multicollinearity and interactions among the study independent variables. We performed the analysis using the statistical software Stata 14 (Stata Corp LP; College Station, TX).

## Results

Of the 1,002 respondents, 811 reported at least one healthcare visit in the last year and hence were eligible for analysis. Ineligible respondents (n = 191) had less education and lower household income; and were less likely to have chronic diseases, social security health insurance, or a usual source of care (S1 Table).

Of the 811 healthcare users, 9% used pharmacy-adjacent doctors, 22.2% used stand-alone private healthcare providers, 43.5% social security providers and 25.3% used other public providers. The four groups did not show statistically significant differences in sex or age (Table 1). However, those attending stand-alone private providers were more likely to have ≥ 10 years of formal than public healthcare providers users. They were also more likely to belong to the middle- or higher-income groups. Almost 40% of private provider users had social security insurance. Only 9% of those using pharmacy-adjacent doctors’ and 25% of those using stand-alone private healthcare providers had private health insurance. Public healthcare users were more likely to have a usual source of care (91%) compared to private provider users (77.4%-80.5%).

**Table 1:** Individual and contextual factors across users of private and public healthcare services in Mexico.

Variable	Type of health services users based on provider used in the last visit					
	Pharmacy-adjacent doctors	Stand-alone private providers	Social Security healthcare providers	Public healthcare providers for those without social security	Total number of observations	
	n = 68	n = 187	n = 373	n = 183	n = 811	
	Weighted N = 69	Weighted N = 171	Weighted N = 335	Weighted N = 194	Weighted N = 769	
	Proportion [95% CI]	Proportion [95% CI]	Proportion [95% CI]	Proportion [95% CI]	Proportion [95% CI]	p-value
	9.0 [6.9, 11.6]	22.2 [19.1, 25.8]	43.5 [39.5, 47.6]	25.3 [21.8, 29.1]	100	
**Socio-demographic factors**						
**I. Individual factors**						
**a. Predisposing attributes**						
Gender						
Women	48.0 [34.8, 61.5]	53.4 [44.8, 61.9]	51.1 [44.8, 57.1]	58.0 [49.2, 66.3]	53.0 [48.9, 57.2]	0.514
Age						0.359
18–44 years	69.5 [55.6, 80.5]	58.6 [50.2, 66.6]	53.2 [47.1, 59.3]	63.4 [55.2, 71.0]	58.5 [54.4, 62.4]	
45–64 years	21.6 [12.3, 35.3]	28.1 [21.5, 35.9]	32.4 [27.0, 38.3]	25.9 [19.5, 33.5]	28.8 [25.4, 32.6]	
65 years or older	8.9 [3.8, 19.3]	13.3 [8.6, 20.0]	14.0 [10.6, 18.02]	9.8 [5.9, 15.9]	12.3 [10.0, 15.0]	
Missing	0	0	0.4 [0.1, 1.7]	0.9 [0.2, 4.2]	0.4 [0.1, 1.2]	
Educational attainment						0.027
High school (≥10years)	45.9 [32.9, 59.4]	55.1 [46.4, 63.5]	46.3 [40.3, 52.3]	36.8 [29.3, 45.1]	45.8 [41.8, 49.9]	
**b. Enabling factors**						
Monthly household income						<0.001
Low income	68.6 [54.6, 79.9]	50.2 [41.8, 58.7]	73.1 [67.7, 78.0]	85.2 [78.5, 90.0]	70.7 [66.9, 74.2]	
Middle and high-income	21.6 [12.3, 35.0]	39.6 [31.6, 48.1]	21.7 [17.3, 26.7]	8.0 [4.7, 13.2]	22.2 [19.1, 25.6]	
Missing	9.8 [4.1, 21.7]	10.2 [5.9, 17.1]	5.2 [3.2, 8.5]	6.9 [3.7, 12.5]	7.2 [5.3, 9.6]	
Social Security health insurance	38.4 [25.6, 53.0]	39.1 [30.9, 47.9]	92.1 [87.9, 94.9]	15.8 [10.5, 23.1]	57.4 [53.1, 61.5]	<0.001
Private health insurance	9.0 [4.0, 19.0]	25.4 [18.3, 34.1]	0	0	6.5 [4.6, 8.9]	<0.001
Usual source of care	77.4 [62.4, 87.7]	80.5 [72.7, 86.4]	91.2 [86.7, 94.3]	91.0 [84.2, 95.0]	87.5 [84.4, 90.1]	0.004
**c. Health needs**						
Fair or poor self-rated health	30.8 [19.9, 44.3]	38.9 [30.9, 47.5]	40.7 [34.9,46.8]	48.9 [40.5,57.3]	41.5 [37.5,45.6]	0.104
Chronic disease	23.9 [13.8, 38.1]	26.9 [20.2, 34.8]	27.0 [22.1, 34.8]	23.6 [17.2, 31.4]	25.8 [22.5, 29.5]	0.859
Unmet health needs in the last year	5.3 [2.0, 13.6]	5.6 [2.7, 11.2]	9.1 [5.9, 13.8]	4.7 [2.4, 9.1]	6.9 [5.0, 9.4]	0.474
**Health services use**						
Number of healthcare visits						0.134
1 visit	18.6 [9.2, 34.0]	17.6 [11.8, 25.5]	13.6 [9.6, 19.0]	14.7 [9.7, 21.0]	15.3 [12.3, 18.7]	
2–3 visits	36.7 [24.6, 50.7]	36.6 [29.1, 44.9]	26.3 [21.2, 32.1]	37.9 [30.1, 46.4]	32.4 [28.7, 36.4]	
≥4 visits	44.7 [31.9, 58.2]	44.1 [35.8, 52.7]	58.7 [52.4, 64.7]	46.8 [38.5, 55.3]	51.2 [47.1, 55.3]	
Not remember/Missing data	0	1.6 [0.5, 5.0]	1.4 [0.7, 3.0]	0.5 [0.1, 3.7]	1.1 [0.6, 2.0]	
Cause of the last visit						<0.001
Urgent or new health problem	82.7 [71.7, 90.0]	47.7 [39.4, 56.2]	42.7 [36.7, 48.9]	42.9 [34.8, 51.4]	47.5 [43.4, 51.6]	
Follow-up care for chronic diseases	8.4 [4.0, 16.8]	14.5 [9.5, 21.6]	24.6 [19.8, 30.1]	23.3 [16.7, 31.6]	20.6 [17.5, 24.1]	
Preventive care or checkup visit	8.9 [4.0, 19.0]	37.5 [29.6, 46.0]	31.5 [26.1, 37.4]	32.6 [25.2, 40.9]	31.1 [27.4, 35.0]	
Missing data	0	0.3 [0.0, 2.0]	1.2 [0.5, 3.0]	1.1 [0.3, 3.7]	0.9 [0.5, 1.8]	
**II. Contextual factors**						
**a. Demographic**						
Area of residence						<0.001
Rural	8.8 [3.9, 18.9]	18.2 [12.2, 26.4]	15.2 [11.1, 25.7]	31.5 [24.0, 40.0]	19.4 [16.2, 23.0]	
Urban	91.2 [81.1, 96.1]	81.5 [73.3, 87.6]	82.7 [77.5, 87.0]	68.2 [59.6, 75.6]	79.5 [75.9, 82.7]	
Missing data	0	0.3 [0.0, 2.1]	2.1 [0.9, 4.8]	0.3 [01, 2.4]	1.1 [0.5, 2.3]	
Region of residence based on average household income						0.009
Poorest	30.0 [19.2, 43.5]	28.4 [21.6, 36.4]	23.7 [19.1, 29.0]	43.0 [35.0, 51.4]	30.2 [26.7, 33.9]	
Middle	28.2 [17.5, 42.1]	33.0 [25.4, 41.6]	41.3 [35.3, 47.7]	29.5 [22.1, 38.1]	35.3 [31.4, 39.5]	
Richest	40.5 [27.8, 54.6]	38.6 [30.6, 47.1]	34.3 [28.8, 40.2]	27.5 [20.4, 35.9]	34.1 [30.3, 38.1]	
Missing	1.3 [0.2, 8.7]	0	0.7 [0.2, 3.4]	0	0.4 [0.1, 1.5]	
**b. Health system**						
Perception of overall quality of the public health sector						<0.001
Excellent/very good/ good	36.6 [24.6, 50.4]	25.3 [18.3, 33.8]	54.7 [48.6, 60.7]	39.9 [32.0, 48.4]	42.8 [38.7, 46.9]	
Fair/Poor	63.4 [49.6, 75.4]	74.7 [66.2, 81.7]	45.3 [39.3, 51.5]	60.1 [51.6, 68.0]	57.2 [53.1, 61.3]	

Concerning health needs, there were no differences in self-rated health. Between 23.6% and 27% of respondents had chronic conditions, and less than 6.9% considered they had unmet health needs in the last year. Healthcare use was high overall: 51.2% reported at least four visits in the last year. For all users, the most frequent reason for the last visit was an urgent or new health problem. The majority of respondents who used doctors adjacent to pharmacies reported using them for an urgent or new health issue (82.7%).

More private and social security users lived in urban areas and in the richest regions, compared to the other public providers users. Public healthcare users were more likely to rate the overall quality of public healthcare services as good to excellent compared to private healthcare users.

**Fig 1:**
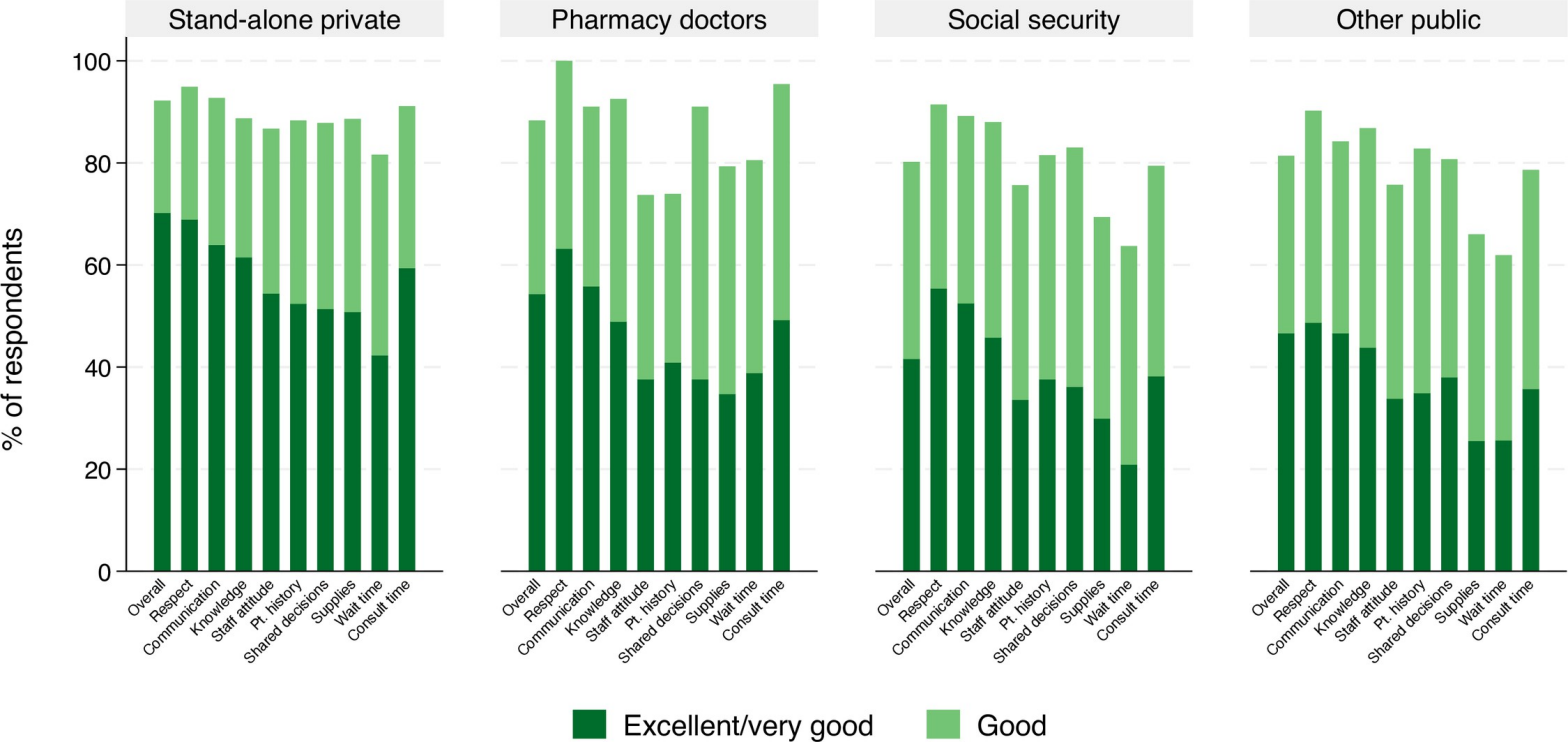
Rating of last visit quality by provider type and quality dimension.

Ratings of most recent visits by provider type are shown in Fig 1 and detailed in S2 Table.

The proportion of users rating the visit as excellent or very good was highest for private providers for overall quality and each quality indicator, including more highly rated items like respectful treatment (57.4% rating excellent or very good overall, 68.9% within private providers) to the lowest rated items for availability of supplies and equipment (33.9% excellent or very good ratings overall, 50.8% within private providers) and wait time (29.4% excellent or very good overall, 42.3% within private providers). Pharmacy-adjacent doctors were rated less positively than private providers, though comparably for wait time and consultation duration. Less than half of the public healthcare users rated their most recent visit as excellent or very good quality overall; public sources were particularly poorly rated for availability of supplies and equipment and wait time.

Perception of overall quality in public health sectors was lower than overall quality of one’s own most recent visit, with 57% of respondents selecting fair or poor for at least one of the three public health systems; all three systems were rated similarly (S3 Table).

The multivariable analysis showed that, after adjusting for other conceptually relevant variables, those with fair or poor perception of overall quality in the public health sector were more likely to seek private healthcare (adjusted incidence rate ratio (aIRR): 1.95; 95% Confidence Intervals: 1.34, 2.82) (Table 2).

**Table 2:** Association between users´ perception of quality in the public health sector with the likelihood of using private healthcare providers (Weighted N = 744).

Variables	Unadjusted IRR	Adjusted IRR
	[95% Conf. Interval]	[95% Conf. Interval]
**Perception of the overall quality of the public health sector as fair or poor**	2.07 [1.48, 2.88]	1.95 [1.34, 2.82]
**Individual predisposing factors**		
Women	1.02 [0.76, 1.37]	1.08 [0.80, 1.45]
Age		
45–64 years	0.84 [0.61, 1.15]	0.91 [0.63, 1.30]
65 years or older	0.81 [0.50, 1.31]	0.92 [0.55, 1.53]
High school or higher (≥10years)	1.41 [1.06, 1.89]	1.17 [0.86, 1.60]
**Individual enabling factors**		
High/middle household income ≥ 10,000 pesos/month	1.92 [1.45, 2.54]	1.74 [1.26, 2.42]
Social security health insurance	0.61 [0.45, 0.83]	0.57 [0.41, 0.79]
Has a regular provider or usual source of care	0.51 [0.37, 0.70]	0.60 [0.42, 0.85]
**Health needs**		
Chronic disease	1.00 [0.72, 1.38]	1.23 [0.84, 1.79]
Fair or poor self-rated health	0.84 [0.63, 1.13]	0.87 [0.60, 1.25]
Unmet need	0.62 [0.32, 1.22]	0.75 [0.39, 1.43]
**Contextual factors**		
Residence in the region with a low average household income	1.23 [0.86, 1.76]	1.22 [0.82, 1.82]
Residence in the region with a high average household income	1.30 [0.91, 1.85]	1.21 [0.83, 1.77]
Residence in an urban area	1.51 [0.99, 2.31]	1.32 [0.81, 2.15]

Results are from a Poisson regression model using stabilized inverse probability weights. Reference categories: men; 18–44 years old; ≤9 years of schooling; low household income <10,000 pesos/month; without social security health insurance; without chronic disease; excellent/very good/good self-rated health; without usual healthcare provider; without unmet healthcare needs; residence in region with middle average household income; residence in rural area.

## Discussion

The present study revealed frequent use of private healthcare services in Mexico, particularly by individuals with high education and income. A substantial proportion of private care users had no private health insurance. These users had access to presumably cheaper options: social security, IMSS-Bienestar or MoH, and other public care sources. Perceived quality was generally higher for private services and even for the pharmacy-adjacent doctors compared to public sources. Additionally, perceiving public health providers as poor quality was associated with a greater probability of private provider use.

Our study found socioeconomic differences between private and public health services users. Previous studies have shown that individuals with lower education, income, and no health insurance in Mexico have poorer health service use and worse health outcomes [20, 33, 34]. Most private healthcare users in Mexico lack private or social security health insurance, reducing their access to essential health services and resulting in high out-of-pocket expenditures when accessing care [20, 35].

Private healthcare users were more likely to rate their healthcare quality highly than public sector users. This finding has been typically reflected in less waiting time and more patient-centered care from private providers [18, 20]. In the present study, the most highly rated characteristics reported by participants attending private healthcare providers were respectful care, clarity of information, and technical knowledge and skills. This perception has been stable over the last decade in Mexico [18–20]. Compared to the National Health and Nutrition Survey (ENSANUT) 2012, our results showed similar users’ perceptions regarding the quality of care across the public and private health sectors. For instance, in 2012, the proportion of people who reported fair or poor quality of care was 11.3% for people who used pharmacy-adjacent doctors, 6.8% for stand-alone private healthcare providers, 22.4% for social security, and 16.9% for MoH [20]. In our study these percentages were 11.7%, 7.8%, 19.8%, and 18.6%, respectively.

Study findings must be situated in the current health policy situation in Mexico. Public health systems in Mexico have experienced substantial upheaval in recent years due not only to the COVID-19 pandemic but also to health policy shifts before and after the severe disruption of this public health emergency [36]. The Seguro Popular program and National Institute of Health for Wellness were terminated in 2019 and 2022, respectively. The subsequent reformulation of IMSS-Bienestar to provide healthcare to the uninsured and the federalization of services provided by state-level MoH secretariats is intended to address the gaps in public healthcare coverage, with unclear progress to date. At the same time, access to healthcare in Mexico remains inadequate, with 35 million people lacking public healthcare insurance coverage in 2021 [10]. Financial protection also remains insufficient even among those with access to insurance: 46.2% of the national health expenditures come from private expenditures, and more than 80% of private expenditures come from out-of-pocket expenditures [10]. Our study adds insights into the substantial deficit in quality of care based on user ratings of private sources *versus* public care and confirms that perceiving public healthcare as of low quality may drive people to use private services, as nearly 40% of private providers users were affiliated with social security institutions. This phenomenon undermines universal access to health services as it contributes to an increase in out-of-pocket expenditures, especially for Social Security beneficiaries who face a dual negative impact, as they not only pay the Social Security payroll tax but also incur out-of-pocket expenses for private healthcare services.

A study in India [37] similarly found that failure to meet users’ expectations can drive patients to private services, even if the clinical competence of private services may be lower. Public health services improvement is critical to increase citizens’ perception of quality and earn their trust in public health institutions, raising public health services use. To achieve this goal, as proposed by the Lancet Global Health Commission on High-Quality Health Systems [27], the Mexican government should strengthen the political commitment to improve the quality of public healthcare services, governing for quality by enacting necessary legislation, investing in the transformation of the health workforce to deliver people-centered high-quality care; and empowering users to demand such care. It is also important to guarantee easy access to the usual source of public health care, which should also provide high-quality services. Having a usual source of care was found to be associated with improved preventive services (cervical cancer screening, clinical breast exam, mammogram, prostate cancer screening, and flu shot) [38] and lower nonurgent emergency department use compared with no usual source of care [39], while in our study having a usual source of care was associated with the lower probability of using private healthcare providers.

The main study limitation is the cross-sectional design that does not allow temporal ordering of study variables; this analysis does not support causal inference. Furthermore, being secondary data analysis, some variables useful to explain health services use are not available, such as users’ functional status and health-related behaviors. Moreover, the study analyzed telephone survey data, which can be prone to social desirability bias when participants provide responses that they think are socially acceptable; however, physical distance between the interviewer and respondent, along with the respondent’s inability to see non-verbal signs, can lead to more sincere responses. Responses may be subject to uncertain recall, although we focused on perceived quality of the most recent healthcare visit to minimize recall time. In addition, the People’s Voice Survey in Mexico had a low response rate. The response rate can be negatively affected by the seasonal effect of conducting survey in December 2022 and January 2023, as some studies have found that survey participation rates tend to be lower during holiday periods [40, 41]. In addition, the growing violence in Mexico and fear of the people to disclose personal information might influence the low response rate [42]. However, it is worth noting that low response rates do not necessarily indicate nonresponse bias. “Nonresponse bias occurs when people who agree to participate in a survey are systematically different from those who refuse to participate” [43]. To ovoid nonresponse bias surveys should use statistical weighting to ensure that their sample is representative of the population with respect to geography, age, gender, education, and other relevant characteristics [43]. In the present study, to overcome the potential selection bias and to ensure that the study results were generalizable to the survey target population we applied survey sampling weights and we also used stabilized IP-weights to address missing information bias.

## Conclusion

Most private healthcare providers’ users lack private health insurance and report a higher quality of healthcare received; the overall perception of public healthcare providers’ low quality is associated with a higher likelihood of private sector use, contributing to high out-of-pocket costs for users and potentially limited commitment to public health system improvement. The findings of this study call for stronger health system governance, focusing on improving access and quality throughout the Mexican public health system through people-centered policies and interventions.

## Supporting information

S1 Table Comparison of socio-demographic characteristics between respondents included and excluded from the analysis. (PDF)

S2 Table Perception of the quality of last healthcare visit across healthcare providers in Mexico. (PDF)

S3 Table Perception of overall quality of public healthcare providers. (PDF)

## Abbreviations

ENSANUT: National Health and Nutrition Survey
IMSS: Mexican Institute of Social Security
LMIC: Low- and middle-income countries
MoH: Ministry of Health
RDD: random digit dialing
