# Supplementary material for: Users’ perception of quality as a driver of private healthcare use in Mexico: Insights from the People’s Voice Survey
Source: PLoS One. 2024 Jun 25;19(6):e0306179. doi: 10.1371/journal.pone.0306179 (PMC11198766; doi:10.1371/journal.pone.0306179)
Supplement: S1 Table — (PDF) [file pone.0306179.s001.pdf]

S1 Table. Comparison of socio-demographic characteristics between respondents included and excluded from the analysis.

Variable	**Analyzed respondents n= 811**	**Excluded respondents n=191**	
**Socio-demographic factors**	Proportion [95% CI]	Proportion [95% CI]	p
**I. Individual factors**			
**a. Predisposing attributes**			
Gender			0.752
Man	47.0 [42.8, 51.1]	48.5 [40.3, 56.7]	
Age			0.917
18 -44 years	58.5 [54.4, 62.4]	57.2 [49.1, 64.9]	
45 - 64 years	28.8 [25.4, 32.6]	31.2 [24.4, 38.8]	
65 years or older	12.3 [10.0, 15.0]	11.1 [7.2, 16.8]	
Missing	0.4 [0.1, 1.2]	0.5 [0.1, 3.7]	
Educational attainment			<0.001
High School or higher (≥10years of schooling)	45.8 [41.8, 49.9]	24.6 [18.1, 31.7]	
**b. Enabling factors**			
Monthly household income			0.002
Low income (< 10,000 pesos/month)	70.7 [66.9, 74.2]	79.8 [73.0, 85.3]	
Middle and high income ≥10,000 pesos/month	22.2 [19.1, 25.6]	10.1 [6.5, 15.4]	
Missing	7.2 [5.3, 9.6]	10.1 [6.2, 15.9]	
Social Security health insurance	57.4 [53.1, 61.5]	41.4 [33.1, 50.2]	0.001
Usual source of care	87.5 [84.4, 90.1]	63.2 [54.7, 90.1]	<0.001
**c. Health needs**			
Fair or poor self-rated health	42.5 [37.5,45.6]	36.4 [28.9, 44.6]	0.270
Chronic disease	25.8 [22.5, 29.5]	15.3 [10.5, 21.9]	0.005
Unmet health need in the last year	6.9 [5.0, 9.4]	6.0 [3.0, 11.6]	0.711
**II. Contextual factors**			
Area of residence			0.021
Rural	19.4 [16.2, 23.0]	29.6 [22.6, 37.8]	
Urban	79.5 [75.9, 82.7]	69.2 [61.1, 76.3]	
Missing data	1.1 [0.5, 2.3]	1.2 [0.3, 4.2]	
Region of residence by average household income			0.739
Poorest	30.2 [26.7, 33.9]	33.2 [26.1, 41.1]	
Middle	35.3 [31.4, 39.5]	32.3 [24.8, 40.7]	
Richest	34.1 [30.3, 38.1]	33.6 [26.3, 41.9]	
Missing	0.4 [0.1, 1.5]	0.9 [0.2, 4.0]	
