# Supplementary material for: Users’ perception of quality as a driver of private healthcare use in Mexico: Insights from the People’s Voice Survey
Source: PLoS One. 2024 Jun 25;19(6):e0306179. doi: 10.1371/journal.pone.0306179 (PMC11198766; doi:10.1371/journal.pone.0306179)
Supplement: S2 Table — (PDF) [file pone.0306179.s002.pdf]

S2 Table. Perception of the quality of last healthcare visit across healthcare providers in Mexico

Variable	Stand-alone private providers n= 187	Pharmacy-adjacent doctors n= 68	Social security healthcare providers n= 373	Public healthcare providers for those without social security n= 183	Total n= 811	
	**Weighted N= 171**	**Weighted N= 69**	**Weighted N= 335**	**Weighted N= 194**	**Weighted N= 769**	
	Proportion [95% CI]	Proportion [95% CI]	Proportion [95% CI]	Proportion [95% CI]		**p**
Overall quality of care of the last consultation*						0.020
Excellent, very good	70.2 [61.7, 77.5]	54.3 [40.5, 67.4]	41.6 [35.7, 47.8]	46.6 [38.3, 55.2]	50.4 [46.3, 54.5]	
Good	22.0 [15.6, 30.0]	34.0 [22.3, 48.0]	38.6 [32.7, 44.8]	34.8 [27.4, 43.0]	33.5 [29.8, 37.5]	
Fair, poor	7.8 [4.2, 14.0	11.7 [5.1, 24.7]	19.8 [15.4, 25.0]	18.6 [12.8, 26.1]	16.1 [13.3, 19.3]	
Respectful healthcare provider						0.014
care	68.9 [60.2, 76.5]	63.2 [48.9, 75.5]	55.4 [49.2, 61.5]	48.7 [40.4, 57.2]	57.4 [53.3, 61.5]	
Excellent, very good	26.0 [18.9, 34.6]	36.8 [24.5, 51.1]	36.0 [30.2, 42.1]	41.5 [33.4, 50.1]	35.2 [31.3, 39.3]	
Good	5.1 [2.3, 10.8]	0	8.6 [5.8, 12.6]	9.8 [5.8, 15.9]	7.3 [5.5, 9.7]	
Fair, poor						
Clarity of information						0.121
Excellent, very good	63.9 [55.2, 71.8]	55.8 [41.8, 68.9]	52.5 [46.3, 58.6]	46.6 [38.2, 55.1]	53.8 [49.7, 57.9]	
Good	28.8 [21.6, 37.64]	35.2 [23.1, 49.6]	36.7 [30.9, 42.8]	37.6 [29.9, 46.0]	35.0 [31.2, 39.1]	
Fair, poor	7.2 [3.7, 13.8]	9.0 [3.4, 21.8]	10.8 [7.8, 14.8]	15.8 [10.6, 22.59]	11.1 [8.8, 13.9]	
Healthcare provider technical knowledge and skills						0.056
Excellent, very good	61.5 [52.7, 69.6]	48.9 [35.6, 62.5]	45.8 [39.8, 52.0]	43.8 [35.6, 52.3]	49.1 [45.0, 53.2]	
Good	27.2 [20.2, 35.6]	43.6 [30.5, 57.6]	42.2 [36.1, 48.5]	43.0 [34.8, 51.5]	39.2 [35.2, 43.3]	
Fair, poor	11.3 [6.5, 19.1]	7.5 [3.1, 17.0]	12.0 [8.7, 16.2]	13.2 [8.5, 20.0]	11.7 [9.3, 14.6]	
Kindness and supportive attitude of the rest of the health facility						<0.001
staff *	54.4 [45.9, 62.7]	37.6 [25.6, 51.5]	33.6 [28.1, 39.7]	33.8 [26.2, 42.3]	38.6 [34.7, 42.7]	
Excellent, very good	32.3 [24.9, 40.7]	36.1 [24.1, 50.3]	42.0 [36.0, 48.2]	41.9 [33.8, 50.3]	39.3 [35.3, 43.4]	
Good	10.0 [5.8, 16.7]	10.5 [4.5, 22.6]	24.4 [19.6, 29.9]	23.4 [17.0, 31.4]	19.7 [16.6, 23.1]	
Fair, poor	3.3 [1.5, 7.3]	15.7 [8.0, 28.4]	0	0.9 [0.2, 4.5]	2.4 [1.4, 3.9]	

The clinic had no other staff						
Health provider knowledge of patients' previous consultations and tests results*						<0.001
Excellent, very good	52.4 [43.9, 60.8]	40.9 [28.3, 54.8]	37.6 [31.8, 43.8]	34.9 [27.3, 43.5]	40.5 [36.6, 44.6]	
Good	35.9 [28.1, 44.4]	33.0 [21.6, 46.8]	43.9 [37.9, 50.1]	47.9 [39.6, 56.3]	42.1 [38.2, 46.2]	
Fair, poor	11.3 [6.7, 18.4]	20.5 [11.3, 34.2]	18.4 [14.1, 23.6]	17.1 [11.6, 24.5]	16.7 [13.8, 20.0]	
Missing	0.4 [0.1, 3.0]	5.6 [1.6, 17.5]	0.1 [0.0, 0.7]	0.1 [0.0, 0.5]	0.7 [0.2, 1.8]	
Patient involvement in making decisions about healthcare						0.053
Excellent, very good	51.4 [42.9, 59.8]	37.6 [25.6, 51.2]	36.1 [30.5, 42.1]	38.0 [30.0, 46.6]	40.1 [36.2, 44.2]	
Good	36.4 [28.6, 45.1]	53.4 [39.8, 66.5]	46.9 [40.7, 53.1]	42.7 [34.6, 51.2]	44.1 [40.0, 48.2]	
Fair, poor	12.2 [7.4, 19.3]	9.0 [3.8, 19.8]	17.0 [12.9, 22.1]	19.3 [13.6, 26.8]	15.8 [13.0, 19.0]	
Availability of equipment and other supplies*						<0.001
Excellent, very good	50.8 [42.3, 59.2]	34.7 [23.1, 48.6]	29.9 [24.4, 36.0]	25.5 [18.7, 33.7]	33.9 [30.1, 37.9]	
Good	37.8 [29.8, 46.4]	44.6 [31.5, 58.5]	39.5 [33.7, 45.6]	40.5 [32.5, 49.1]	39.8 [35.8, 43.9]	
Fair, poor	10.6 [6.3, 17.3]	17.8 [9.9, 29.7]	29.3 [24.1, 35.0]	33.0 [25.7, 41.3]	25.0 [21.7, 28.7]	
Missing	0.9 [0.1, 5.9]	2.9 [0.4, 17.9]	1.3 [0.5, 3.6]	1.0 [0.2, 4.7]	1.3 [0.6, 2.7]	
Wait time*						<0.001
Excellent, very good	42.3 [34.2, 50.8]	38.8 [26.4, 52.7]	20.9 [16.2, 26.5]	25.6 [18.9, 33.7]	29.4 [24.9, 32.3]	
Good	39.3 [31.4, 47.8]	41.7 [29.0, 55.6]	42.8 [36.8, 49.0]	36.3 [28.6, 44.7]	40.3 [36.3, 44.4]	
Fair, poor	18.4 [12.3, 26.7]	17.4 [9.3, 30.4]	35.7 [30.1, 41.7]	38.1 [30.2, 46.7]	30.8 [27.2, 34.8]	
Missing	0	2.1 [0.3, 13.6]	0.6 [0.1, 3.1]	0	0.5 [0.1, 1.6]	
Consultation time *						<0.001
Excellent, very good	59.4 [50.8, 67.5]	49.2 [35.8, 62.7]	38.2 [41.2, 44.3]	35.7 [28.1, 44.2]	43.3 [39.3, 47.4]	
Good	31.7 [24.2, 40.3]	46.2 [32.9, 59.9]	41.2 [35.3, 47.5]	42.9 [34.8, 51.4]	40.0 [36.0, 44.1]	
Fair, poor	8.9 [5.2, 14.9]	4.6 [1.5, 13.8]	20.5 [16.0, 25.9]	21.4 [15.1, 29.4]	16.7 [13.9, 20.0]	

*p<0.05
