# Supplementary material for: Users’ perception of quality as a driver of private healthcare use in Mexico: Insights from the People’s Voice Survey
Source: PLoS One. 2024 Jun 25;19(6):e0306179. doi: 10.1371/journal.pone.0306179 (PMC11198766; doi:10.1371/journal.pone.0306179)
Supplement: S3 Table — (PDF) [file pone.0306179.s003.pdf]

S3 Table. Perception of overall quality of public healthcare providers

	Social Security	Ministry of Health	IMSS Bienestar
	Proportion [95% CI]	Proportion [95% CI]	Proportion [95% CI]
Excellent/ Very good	21.7 [18.3, 25.5]	20.0 [16.8, 23.7]	21.1 [17.7, 24.9]
Good	35.1 [31.2, 39.2]	39.2 [35.1, 43.4]	37.7 [33.7, 41.9]
Fair/ Poor	42.1 [38.0, 46.3]	38.7 [34.6, 42.8]	37.5 [33.5, 41.7]
Missing data	1.1 [0.5, 2.3]	2.1 [1.2, 3.7]	3.7 [2.5, 5.3]
